# Immune Monitoring Technology Primer: protein microarray (‘seromics’)

**DOI:** 10.1186/s40425-016-0106-4

**Published:** 2016-01-19

**Authors:** Jianda Yuan, Ena Wang, Bernard A. Fox

**Affiliations:** Immune Monitoring Core, Ludwig Center for Cancer Immunotherapy, Memorial Sloan Kettering Cancer Center, 1275 York Ave, Box 386, New York, NY 10065 USA; Sidra Medical and Research Center, PO Box 26999, Doha, Qatar; Earle A. Chiles Research Institute, Providence Cancer Center, 4805 NE Glisan Street, Portland, OR 97213 USA

**Keywords:** Immune monitoring, Protein microarray, Biomarker, Seromics, Autoantibody response

## Description of the technology

Protein microarrays are used to identify targets of drugs, small molecules, and enzyme substrates that may be modified in numerous ways such as by phosphorylation or methylation, to detect protein binding properties, investigate protein-protein interactions, and to define protein-based biomarkers in a high-throughput manner. They are based on the development of DNA microarray techniques and have thousands of purified proteins immobilized on a solid surface. The technique requires a blocking step to be applied to remove any non-specific binding. After incubation of the protein probe (e.g. patient serum) with a suitable tag, visible detection reagents are employed to quantify the corresponding signals. Proteins microarray are classified into three types: analytical, functional and reverse-phase protein microarrays [1]. Functional protein microarrays are widely used because of their commercial availability. As an example, ProtoArray® offers a unique approach to analyze the serological response against thousands of proteins (~9000) simultaneously. ProtoArray® does not cover the entire proteome, but a serological analysis of clinical samples using this protein microarray allows high-content "seromics” screening against a substantial portion of the human proteome to identify potential antibody biomarkers and reactivity patterns that may correlate with disease state or treatment response.

A new, emerging protein array that utilizes molecular technology for the detection of protein abundance in serum, other body fluids and cell lysates. This technology is represented by SomaLogic's SOMAmer® (Slow Off-rate Modified Aptamer) proteomic assay. This technology combines the properties of antibodies and traditional aptamers with extremely large specificity assembly. The selected target specific aptamers with specific three-dimensional structure are modified into a “SOMAmer” that provides protein-binding specificity as well as the primary nucleic acid sequence backbones with streptavidin linker attached to beads bind to proteins in the sample mix. The proteins that are bound to their specific SOMAmer reagents are then biotinylated followed by photocleaving of the linker to release the SOMAmer-protein complexes. Via subsequent capture, all biotinylated proteins are bound to a second streptavidin bead followed by denature of SOMAmer-protein targets, protein targeted SOMAmer are collected, labeled, denatured and hybridized to DNA array fabricated by the complementary strand of the SOMAmers. This technology can efficiently, accurately and rapidly identify and quantify over 1000 proteins across approximately eight logs of concentration in small sample volumes. Other protein microarrays are under research level development and not yet widely applied for immune monitoring.

**Fig. 1:**
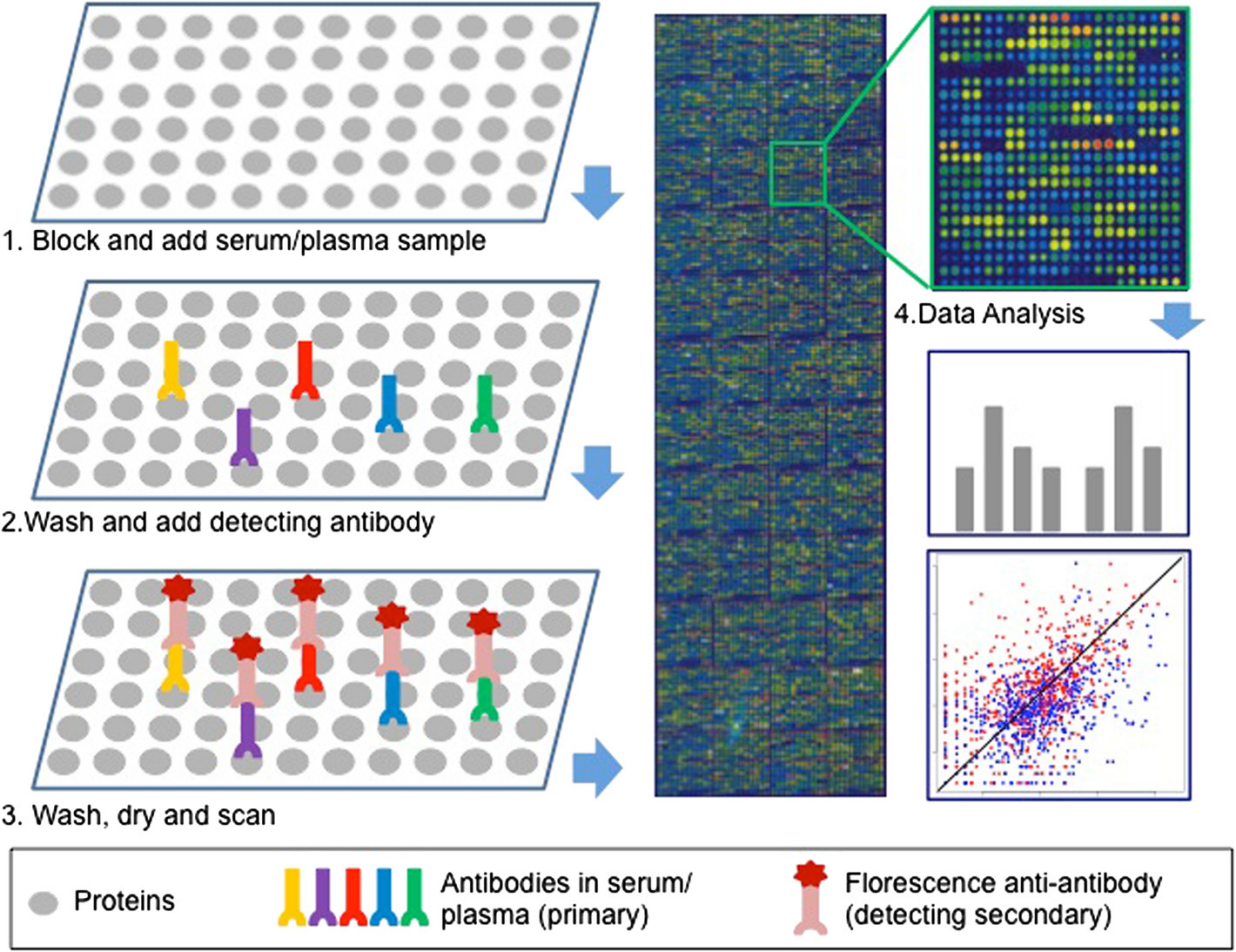
Workflow of protein microarray development for the antibody response biomarker profiling. Step 1: Incubate the slides with blocking buffer to reduce non-specific background, then add diluted serum/plasma samples for two hours’ incubation. Step 2: Wash the slides with probing/washing buffer, then incubate fluorescence conjugated anti-human IgG antibody for another two hours. Step 3: Wash slides with probing buffer and deionized water, and dry the slides with centrifugation. After drying, immediately transfer the arrays to a slide carrier of the GenePix 4200AL fluorescent microarray scanner. Step 4: Scan the array, and analyze the data in GenePix Pro 6.1 software or export the results for additional analysis

Here, we concentrate on the ‘seromics’ ProtoArray**®** technology for the following discussion regarding samples and data in detail. The workflow of an example protein microarray is illustrated in Fig. 1, showing the approach for detecting an antibody response in peripheral blood by this platform [2, 3].

### Type of data obtained/readout

Similar to DNA microarrays, a potentially large number of fluorescence signals may be obtained from each array depending on the amount and diversity of antibodies or proteins in each sample that may bind to the thousands of proteins coated on each slide. The results of positive controls are used to determine the quality of the assay. A microarray scanner is required to read and quantitate the fluorescence units from each slide, and results can be exported into spreadsheet format. Bioinformatics is essential to handling and processing the large amount of data obtained through multiple steps including data acquisition, pre-processing, visualization, differential analysis, result verification and computational feature annotation and network analysis. Several software and computational tools have been developed for signal detection, data preprocessing, quality control and data normalization [4–6].

### Limitations of the approach

Different proteins may be coated at different amounts on the slides resulting in potentially different levels of fluorescence signal; therefore the assay is not meant to be quantitative when comparing results between the various proteins. As may be expected from high content analyses, there are also potential non-specific, false positive events that can be found from protein microarray analysis. Therefore, it is critical that the identified antibodies/antigens should be validated by other assays such as western blot, ELISA, Luminex or mass spectrometry. Given the large scale of protein synthesis required for manufacture of the arrays and the ability to run only 1 sample per slide, the assay is relatively expensive which needs to be overcome for more widespread application and use in larger scale clinical trials. The somewhat limited number of proteins coated on the slides (e.g. not all human proteins are present and mutated or modified versions of proteins may not be available), and lack of availability of antibodies in other complimentary platforms to verify specificity of each of the proteins on the slides also currently restricts the broader application of the analytical protein microarray platform.

### Types of samples needed and special issues pertaining to samples

Proper collection and storage of samples and careful standardized sample processing procedure are required to minimize inter- and intra-assay variation and improve the data reproducibility. Frozen serum and plasma samples collected from blood are usually kept in −20 or −80 °C freezer for long-term storage to preserve sample stability. Batched pre- and post-therapy sample analyses performed in a single run are recommended to avoid possible differences arising from the inter-assay variation. Samples should not be subjected to multiple freeze-thaw cycles in order to minimize potential degradation of proteins in the sample as well. Special attention should be given to therapeutic agents that may be present in samples as a result of patient drug therapy which may interfere with interpretation of results due to interactions with proteins in the arrays.

### Level of evidence

The sensitivity and specificity of a protein microarray were compared to the standard ELISA with a 94 % concordance [2, 3]. Specific autoantibody responses have been associated with tumor progression for patients with a wide range of cancers [7–9]. Humoral antigen spreading response induced by Sipuleucel-T therapy has been shown to be associated with improved overall survival [10]. Cytotoxic T lymphocyte antigen-4 (CTLA-4) blockade was showed to induce a larger number of antibody responses in the clinical responders than non-responders in patients with prostate cancer [6]. It was also shown that a prostate cancer patient with a sustained complete response to CTLA-4 blockade mounted a strong humoral response against a small number of proteins [11]. A major question for this technology is whether the development of IgG antibody responses, which require CD4 T cell help for Ig class-switching, can serve as a surrogate for T cell immunity [4]. More than 150 papers have been published using this ProtoArray**®** platform in last ten years. Further research is warranted to validate this novel technology and these potential biomarkers sufficiently in the clinical setting for routine clinical application.

## Abbreviations

SOMAmer: Slow Off-rate Modified Aptamer
DNA: Deoxyribonucleic acid
ELISA: Enzyme-linked immunosorbent assay
CTLA-4: Cytotoxic T lymphocyte antigen-4
IgG: Immunoglobulin G.
